# First report of putative *Leishmania* RNA virus 2 (LRV2) in *Leishmania infantum* strains from canine and human visceral leishmaniasis cases in the southeast of Brazil

**DOI:** 10.1590/0074-02760230071

**Published:** 2023-09-18

**Authors:** Felipe Dutra Rêgo, Eduardo Sérgio da Silva, Valeriana Valadares Lopes, Rafael Gonçalves Teixeira-Neto, Vinícius Silva Belo, Antônio Augusto Fonseca, Diego Andrade Pereira, Heber Paulino Pena, Márcia Dalastra Laurenti, Gabriela V Araújo, Vânia Lúcia Ribeiro da Matta, Islam Hussein Chouman, Thainá Bergantin Burrin, Carmen M Sandoval, Stella Maria Barrouin-Melo, Flaviane Alves de Pinho, Hélida Monteiro de Andrade, Ramon Vieira Nunes, Célia Maria Ferreira Gontijo, Vanete Thomaz Soccol, Donnamae Klocek, Danyil Grybchuk, Diego Henrique Macedo, Rubens Lima do Monte-Neto, Vyacheslav Yurchenko, Rodrigo Pedro Soares

**Affiliations:** 1 Fundação Oswaldo Cruz-Fiocruz, Instituto René Rachou, Grupo de Pesquisa em Biotecnologia Aplicada ao Estudo de Patógenos, Belo Horizonte, MG, Brasil; 2 Universidade Federal de São João Del Rei, Laboratório de Doenças Parasitárias e Infecciosas, Divinópolis, MG, Brasil; 3 Ministério da Agricultura, Pecuária e Abastecimento, Laboratório Nacional Agropecuária, Pedro Leopoldo, MG, Brasil; 4 Universidade de São Paulo, Faculdade de Medicina, Departamento de Patologia, Laboratório de Patologia de Moléstias Infecciosas, São Paulo, SP, Brasil; 5 Universidade Federal da Bahia, Departamento de Anatomia, Patologia e Clínicas Veterinárias, Escola de Medicina Veterinária, Laboratório de Infectologia Veterinária, Salvador, BA, Brasil; 6 Universidade Federal de Minas Gerais, Instituto de Ciências Biológicas, Departamento de Parasitologia, Laboratório de Leishmanioses, Belo Horizonte, MG, Brasil; 7 Universidade Federal do Paraná, Departamento de Engenharia de Bioprocessos e Biotecnologia, Curitiba, PR, Brasil; 8 University of Ostrava, Faculty of Science, Life Science Research Centre, Ostrava, Czech Republic

**Keywords:** Leishmania, Leishmania infantum, leishmaniasis, *Leishmania* RNA virus, Totiviridae

## Abstract

**BACKGROUND:**

*Leishmania* RNA virus 1 (LRV1) is commonly found in South American *Leishmania* parasites belonging to the subgenus *Viannia*, whereas *Leishmania* RNA virus 2 (LRV2) was previously thought to be restricted to the Old-World pathogens of the subgenus *Leishmania*.

**OBJECTIVES:**

In this study, we investigated the presence of LRV2 in strains of *Leishmania (L.) infantum*, the causative agent of visceral leishmaniasis (VL), originating from different hosts, clinical forms, and geographical regions.

**METHODS:**

A total of seventy-one isolates were screened for LRV2 using semi-nested reverse transcription-polymerase chain reaction (RT-PCR) targeting the RNA-dependent RNA polymerase (RdRp) gene.

**FINDINGS:**

We detected LRV2 in two *L. infantum* isolates (CUR268 and HP-EMO) from canine and human cases, respectively.

**MAIN CONCLUSIONS:**

To the best of our knowledge, this is the first detection of LRV2 in the New World.

*Leishmania* (Kinetoplastea: Trypanosomatidae) are protistan parasites that cause leishmaniasis, a vector-borne disease prevalent in nearly 100 countries.^1^ ^,^ ^2^ This disease exhibits a wide range of clinical manifestations resulting from complex interactions between the parasite and the host immune system.^3^ ^,^ ^4^ However, despite the substantial efforts to elucidate the molecular mechanisms governing this intricate interface, they remain poorly understood. The presence of endosymbiotic *Leishmania* viruses has been suggested as an important component in this puzzle.^5^ ^,^ ^6^ ^,^ ^7^

Various representatives of the subgenera *Leishmania*, *Mundinia*, and *Viannia* have been screened for the presence of the virus, leading to the discovery of double-stranded *Leishmania* RNA viruses (LRVs, family *Totiviridae*) and negative-sense single-stranded RNA viruses, known as leishbuviruses (LBV, family *Leishbuviridae*, older name *Leishbunyaviridae*).^8^ ^,^ ^9^ ^,^ ^10^ LRVs encompass four species, namely LRV1 to LRV4. LRV1 and LRV2 have been found primarily in the subgenus *Viannia* (*L. braziliensis*, *L. guyanensis*, *L. lainsoni*, *L. naiffi*, *L. panamensis*, and *L. shawi*), and the subgenus *Leishmania* (*L. aethiopica*, *L. infantum*, *L. major*, and *L. tropica*), respectively.^9^ ^,^ ^10^ ^,^ ^11^ ^,^ ^12^ The other two species, LRV3 and LRV4, have been documented in a different trypanosomatid group known as the genus *Blechomonas*.^13^ LBV representatives have been found to infect *L. martiniquensis*, of the subgenus *Mundinia*, in Martinique and Brazil.^14^ ^,^ ^15^

Despite recent advancements in the field, the extent to which the virus affects parasite virulence or host immune responses during *Leishmania* infection remains unclear. In the case of LRVs, it is known that the endosymbiotic virus confers an adaptive advantage to the parasite by suppressing anti-leishmanial immunity in the vertebrate host, thereby facilitating the parasite’s survival.^6^ ^,^ ^16^ The exacerbation of the LRV1-mediated disease appears to be dependent on TLR3 limiting the activation of the NLRP3 inflammasome in macrophages, resulting in elevated parasitaemia and destructive mucosal inflammation.^5^ ^,^ ^17^ ^-^ ^20^ LRV2 from *L. aethiopica* also modulates the host immune response, leading to a TLR3-dependent hyperinflammatory response.^11^ Additionally, LRV1 can be shed *via* exosomes and this process may also govern *Leishmania* virulence.^21^ ^,^ ^22^ ^,^ ^23^ Interestingly, LRV1 and LRV2 may elicit different responses in various *Leishmania* species.^24^

While dsRNA viruses have been detected in various *Leishmania* spp., their prevalence varies among isolates of a given species.^25^ In the New World, LRV1s are predominant in clinical isolates from Bolivia, Colombia, Ecuador, French Guiana, Peru, and Suriname,^12^ ^,^ ^26^ ^,^ ^27^ ^,^ ^28^ while being rare in southeastern Brazil.^29^ ^,^ ^30^ LRV2 has been detected in Central Asian and Middle Eastern countries, with a higher prevalence in *L. major* compared to other susceptible species.^11^ ^,^ ^31^ ^-^ ^35^ Interestingly, LRV2-positive strains have not yet been detected in the New World, despite the presence of certain Old-World species of the subgenus *Leishmania*, known to be virus-positive in Asia. This suggests the independent co-evolution of LRV1 and LRV2 with their hosts in the New and Old Worlds, respectively.^9^ ^,^ ^10^ ^,^ ^36^ In this work, we analysed *L. (L.) infantum* strains from Brazil and Honduras for the presence of LRV2.

## MATERIALS AND METHODS

**Fig. 1:**
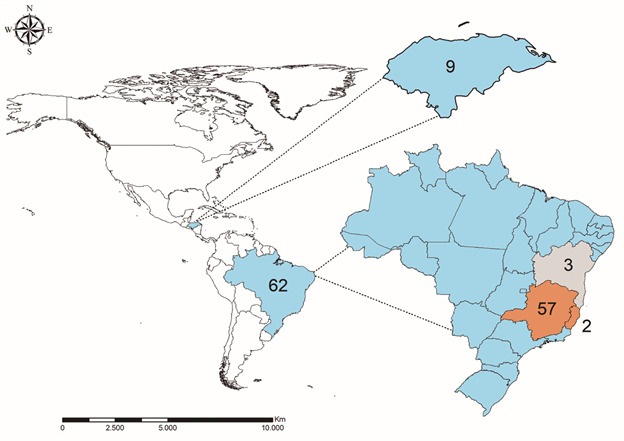
origin (Honduras and Brazil) and number of New World strains of *Leishmania infantum* analysed in this study for the presence of *Leishmania* RNA virus 2 (LRV2). Brazilian states marked in orange indicate LRV2 positive strains.

*Parasite culture, DNA and RNA isolation, and complementary DNA (cDNA) synthesis* - A panel comprising 71 strains of *L. infantum*, including World Health Organization reference strains and field isolates (Fig. 1, Table), was initially cultured in M199 medium supplemented with 10% foetal bovine serum (FBS) (Thermo Fisher Scientific, Waltham, USA), penicillin (200 u/mL), and streptomycin (200 μg/mL) (all from Merck, Darmstadt, Germany) at 25ºC for one to three weeks. DNA was extracted from 5 × 10^7^ cells using the PureGene Core Kit A (Qiagen, Hilden, Germany). The *hsp70* gene was amplified and digested with *Hae*III to confirm the parasite’s identity^37^ [Supplementary data (Figure)].

**TABLE:** New World *Leishmania* (*Leishmania*) *infantum* strains prospected in this study for the presence of *Leishmania* RNA virus 2 (LRV2)

Sample	Country (State)	Clinical form	LRV2
MHOM/BR/2001/HP-EMO	Brazil (Espírito Santo)	VL	+
MCAN/BR/2004/CUR268	Brazil (Minas Gerais)	CVL	+
MHOM/HN/2017/AM-65	Honduras (Valle)	NUCL	-
MHOM/HN/2017/AM-73	Honduras (Valle)	NUCL	-
MHOM/HN/2018/AMA-161	Honduras (Valle)	NUCL	-
MHOM/HN/2018/AMA-614	Honduras (Valle)	NUCL	-
MHOM/HN/2019/AMA-900	Honduras (Valle)	NUCL	-
MHOM/HN/2019/AMA-901	Honduras (Valle)	NUCL	-
MHOM/HN/2019/AMA-902	Honduras (Valle)	NUCL	-
MHOM/HN/2019/AMA-904	Honduras (Valle)	NUCL	-
MHOM/HN/2020/LV-3	Honduras (Lempira)	VL	-
MCAN/BR/1999/JP15	Brazil (Minas Gerais)	CVL	-
MCAN/BR/2016/52	Brazil (Minas Gerais)	CVL	-
MCAN/BR/2016/57	Brazil (Minas Gerais)	CVL	-
MCAN/BR/2016/90	Brazil (Minas Gerais)	CVL	-
MCAN/BR/2016/91	Brazil (Minas Gerais)	CVL	-
MCAN/BR/2016/121	Brazil (Minas Gerais)	CVL	-
MCAN/BR/2016/138	Brazil (Minas Gerais)	CVL	-
MCAN/BR/2016/140	Brazil (Minas Gerais)	CVL	-
MCAN/BR/2016/164	Brazil (Minas Gerais)	CVL	-
MCAN/BR/2016/177	Brazil (Minas Gerais)	CVL	-
MHOM/BR/2019/RdM-JVJ	Brazil (Minas Gerais)	VL	-
MHOM/BR/1974/PP75	Brazil (Bahia)	VL	-
MHOM/BR/1989/Ba262	Brazil (Bahia)	VL	-
MFEL/BR/2018/CAT1	Brazil (Bahia)	FVL	-
MHOM/BR/1987/HCO-1	Brazil (Espírito Santo)	VL	-
MHOM/BR/1970/BH46	Brazil (Minas Gerais)	VL	-
Isolate 75 (UFMG)	Brazil (Minas Gerais)	CVL	-
Isolate 76 (UFMG)	Brazil (Minas Gerais)	CVL	-
Isolate 82 (UFMG)	Brazil (Minas Gerais)	CVL	-
Isolate 83 (UFMG)	Brazil (Minas Gerais)	CVL	-
Isolate 84 (UFMG)	Brazil (Minas Gerais)	CVL	-
Isolate 94 (UFMG)	Brazil (Minas Gerais)	CVL	-
Isolate 97 (UFMG)	Brazil (Minas Gerais)	CVL	-
Isolate 99 (UFMG)	Brazil (Minas Gerais)	CVL	-
Isolate 107 (UFMG)	Brazil (Minas Gerais)	CVL	-
Isolate 88 (UFMG)	Brazil (Minas Gerais)	CVL	-
Isolate 101 (UFMG)	Brazil (Minas Gerais)	CVL	-
Isolate 106 (UFMG)	Brazil (Minas Gerais)	CVL	-
MCAN/BR/2019/RR545	Brazil (Minas Gerais)	CVL	-
MCAN/BR/2019/RR546	Brazil (Minas Gerais)	CVL	-
MCAN/BR/2019/RR547	Brazil (Minas Gerais)	CVL	-
MCAN/BR/2019/RR548	Brazil (Minas Gerais)	CVL	-
MCAN/BR/2019/RR549	Brazil (Minas Gerais)	CVL	-
MCAN/BR/2019/RR500	Brazil (Minas Gerais)	CVL	-
MCAN/BR/2019/RR501	Brazil (Minas Gerais)	CVL	-
MCAN/BR/2019/RR516	Brazil (Minas Gerais)	CVL	-
MCAN/BR/2019/RR515	Brazil (Minas Gerais)	CVL	-
MCAN/BR/2019/RR510	Brazil (Minas Gerais)	CVL	-
MCAN/BR/2019/RR507	Brazil (Minas Gerais)	CVL	-
MCAN/BR/2014/UFSJ-DIV-01	Brazil (Minas Gerais)	CVL	-
MCAN/BR/2014/UFSJ-DIV-02	Brazil (Minas Gerais)	CVL	-
MCAN/BR/2014/UFSJ-BRU-19	Brazil (Minas Gerais)	CVL	-
MCAN/BR/2014/UFSJ-BRU-20	Brazil (Minas Gerais)	CVL	-
MCAN/BR/2014/UFSJ-BRU-22	Brazil (Minas Gerais)	CVL	-
MCAN/BR/2014/UFSJ-BRU-25	Brazil (Minas Gerais)	CVL	-
MCAN/BR/2014/UFSJ-ITA-36	Brazil (Minas Gerais)	CVL	-
MCAN/BR/2014/UFSJ-ITA-37	Brazil (Minas Gerais)	CVL	-
MCAN/BR/2014/UFSJ-ITA-39	Brazil (Minas Gerais)	CVL	-
MCAN/BR/2014/UFSJ-ITA-40	Brazil (Minas Gerais)	CVL	-
MCAN/BR/2014/UFSJ-ITA-41	Brazil (Minas Gerais)	CVL	-
MCAN/BR/2014/UFSJ-ITA-42	Brazil (Minas Gerais)	CVL	-
MCAN/BR/2014/UFSJ-ITA-44	Brazil (Minas Gerais)	CVL	-
MCAN/BR/2014/UFSJ-ITA-46	Brazil (Minas Gerais)	CVL	-
MCAN/BR/2014/UFSJ-ITA-47	Brazil (Minas Gerais)	CVL	-
MCAN/BR/2014/UFSJ-ITA-49	Brazil (Minas Gerais)	CVL	-
MCAN/BR/2014/UFSJ-ITA-50	Brazil (Minas Gerais)	CVL	-
MCAN/BR/2014/UFSJ-ITA-54	Brazil (Minas Gerais)	CVL	-
MCAN/BR/2014/UFSJ-ITA-55	Brazil (Minas Gerais)	CVL	-
MCAN/BR/2014/UFSJ-ITA-5	Brazil (Minas Gerais)	CVL	-
MCAN/BR/2004/CUR211	Brazil (Minas Gerais)	CVL	-

NUCL: non-ulcerated cutaneous leishmaniasis; VL: visceral leishmaniasis; CVL: canine visceral leishmaniasis; FVL: feline visceral leishmaniasis; +: LRV2 positive strain; -: LRV2 negative strain.

RNA was isolated from 1 x 10^8^ cells using the Trizol method according to the manufacturer’s protocol (Thermo Fisher Scientific). One hundred ng of each RNA sample was treated with DNAse I prior to complementary DNA (cDNA) synthesis. Reverse transcription was performed using the Super Script III- First Strand Synthesis Kit (Invitrogen), with random hexamer primers according to the manufacturer’s specifications.

*Detection of Leishmania RNA virus 2 and sequencing* - Semi-nested reverse transcription-polymerase chain reaction (RT-PCR) was performed to target the RNA-dependent RNA polymerase (RdRp) gene. In the first stage, a 526 bp fragment was amplified using the primers LRVF-HR (5′-TGTAACCCACATAAACAGTGTGC-3′) and LRVR-HR (5′-ATTTCATCCAGCTTGACTGGG -3′). In the second stage, a 315-bp internal fragment of RdRp was amplified using the primers LRVF2-HR (5′-AGGACAATCCAATAGGTCGTGT-3′) and LRVR-HR.^32^ To ensure cDNA integrity, β-tubulin locus was used as a positive control. It was amplified using the primers bTUBf (5′-ACTGGATCCATGCGTGAGATCGTTTCCTGCC-3′) and BtubR (5′-GACAGATCTCATCAAGCACGGAGTCGATCAGC-3′).^11^ In all experiments, cDNA from the reference LRV2-positive strain of *L. major* (MHOM/SU/1973/5-ASKH) was used as a positive control. PCR products were purified and sequenced according to the manufacturers’ protocols.

*Sequence analysis and phylogenetic inference* - Partial RdRp sequences obtained from the positive isolates (HP-EMO, 305 nt and CUR268, 293 nt) were used as queries in BLASTN searches against the NCBI nt database, resulting in 52 LRV2 sequences. These were aligned iteratively using an MAFFT v. 7.511 program (g-INS-i algorithm).^38^ The alignment was used directly (without trimming) for phylogenetic inference using an IQTree2 program with an automatically selected model (GTF + F + I + G4) and 1,000 thorough bootstrap replicas for statistical support.^39^ The LRV2 from Ethiopia were chosen as the closest outgroup based on previous studies.^11^ ^,^ ^34^ Tree rendering was done using MEGA11.^40^ The RdRp sequences obtained in this study were deposited in the GenBank database under accession numbers OR208212 and OR208213.

## RESULTS

**Fig. 2:**
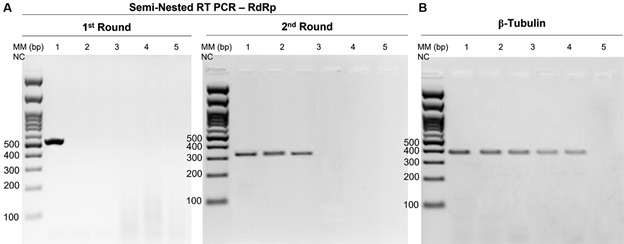
detection of *Leishmania* RNA virus 2 (LRV2) in *L. infantum* isolates by targeting the RNA-dependent RNA polymerase (RdRp) encoding gene. (A) 526 bp and 315 bp amplicons from the first and second round, respectively, of semi-nested amplification. (B) 396 bp product of the β-tubulin encoding gene reverse transcription-polymerase chain reaction (RT-PCR) assessing the integrity of cDNA synthesis. Lanes: MM, molecular weight marker (100 bp); 1, LRV2+ strain of *L. major* (MHOM/SU/1973/5-ASKH); 2 and 3, *L. infantum* strains positive for LRV2 (MCAN/BR/2004/CUR268 and MHOM/BR/2001/HP-EMO, respectively); 4 and 5, *L. infantum* negative strains for LRV2 (MHOM/BR/1970/BH46 and MHOM/BR/1989/Ba262, respectively); NC, non-template control.

**Fig. 3:**
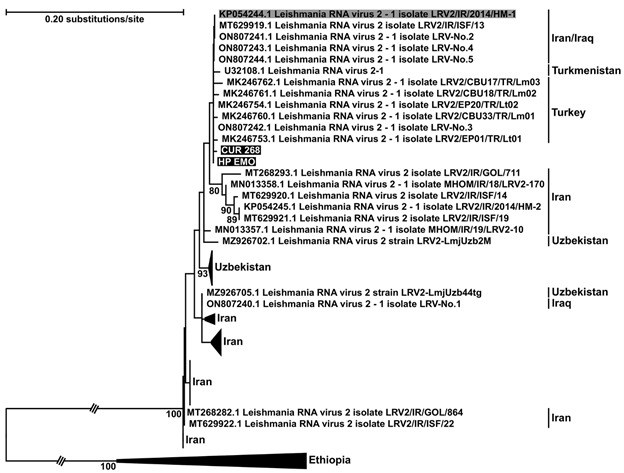
phylogenetic tree based on nucleotide sequences of *Leishmania* RNA virus 2 (LRV2) viruses isolated in different countries. Clades were collapsed for better visibility with the number of samples indicated in brackets. Numbers at branches are thorough bootstrap supports, values smaller than 75 were hidden, dots indicate absolute support (100). Sequences reported in this study are highlighted in black. LRV2 virus isolated from *L. infantum* in Iran is highlighted in grey. Branches with triple crossing were contracted three times of their actual length. Tree was rooted on mid-point.

Out of the 71 samples analysed, two *L. infantum* isolates (CUR-268 and HP-EMO) were found to be positive for the 315 bp fragment at the 3′-end of RdRp using semi-nested RT-PCR analysis (Fig. 2). The resulting sequences showed a high degree of similarity to previously described LRV2 isolates from various regions in Asia (Fig. 3). Specifically, the sequences of both isolates were identical to LRV2 isolated from *L. tropica* in Turkey (isolate LRV2/EP01/TR/Lt01), with only a single nucleotide substitution compared to LRV2 from *L. infantum* in Iran (isolate LRV2/IR/2014/HM-1). A slightly higher degree of divergence (2-7 substitutions) was observed when compared with LRV2 sequences from Uzbekistan.^34^ Phylogenetic analysis placed both isolates within the Asian clade of LRV2. However, due to the lack of complete genomic sequences for most isolates, it is challenging to determine the precise relationships between the viruses within this clade. All strains tested positive for β-tubulin (Fig. 2).

## DISCUSSION

Endosymbiotic double-stranded RNA viruses (dsRNA) have been identified in various protistan parasites and the presence of LRVs in *Leishmania* spp. is considered one of the major factors associated with the severity of leishmaniasis.^5^ ^,^ ^6^ ^,^ ^41^ In this study, we report the first detection of two putative LRV2 strains of *L. infantum* in Brazil. These strains were isolated from cases of VL in dogs and humans in the states of Minas Gerais and Espírito Santo, respectively, in southeastern Brazil. Until now, LRV2 has been detected in *L. aethiopica*,^11^, *L. major*,^31^ ^,^ ^33^ Old World *L. infantum* ^32^ and *L. tropica*.^42^ This finding represents the first report of LRV2 in the New World.

Our analysis revealed only two LRV2-positive strains (2.8%) out of 71 samples analysed. The low frequency of LRV2 detection could be influenced by a variety of factors, including the potential impact of successive *in vitro* passages on the presence and stability of the endosymbiotic virus.^24^ ^,^ ^43^ ^,^ ^44^ In this study, a panel of *L. infantum* strains was used to assess the presence of LRV2, and this may have contributed to the limited detection of the endosymbiotic virus. However, the low frequency observed in our study is in line with the overall prevalence of LRV in Brazil, suggesting that LRV2 is indeed less common in this region. LRV1 is more frequently found in the Amazon Basin (Northern region) and less common in the other regions.^29^ ^,^ ^45^ In southeastern Brazil, only a few cases of LRV1 have been documented, including biopsies of patients with cutaneous leishmaniasis in the municipality of Caratinga, Minas Gerais^46^ and in Rio de Janeiro.^30^ ^,^ ^47^

In our study, the Brazilian LRV2 isolates showed a high degree of similarity with LRV2 isolates from various parts of Asia. However, it is important to interpret this result with caution due to the relatively small size of the amplified fragment (~300 bp). Therefore, a comprehensive analysis of the whole genome would be desirable, to determine the phylogenetic relationships accurately. We believe that our study contributes to the understanding of the geographical distribution of LRV2s in *Leishmania* (*Leishmania*) and provides new avenues for investigating the evolutionary relationships between LRV-infected *Leishmania* parasites and their vertebrate hosts.
