# Supplementary material for: First report of putative Leishmania RNA virus 2 (LRV2) in Leishmania infantum strains from canine and human visceral leishmaniasis cases in the southeast of Brazil
Source: Mem Inst Oswaldo Cruz. 2023 Sep 18;118:e230071. doi: 10.1590/0074-02760230071 (PMC10511063; doi:10.1590/0074-02760230071)
Supplement: Supplementary file 1 [file 1678-8060-mioc-118-e230071-s.pdf]

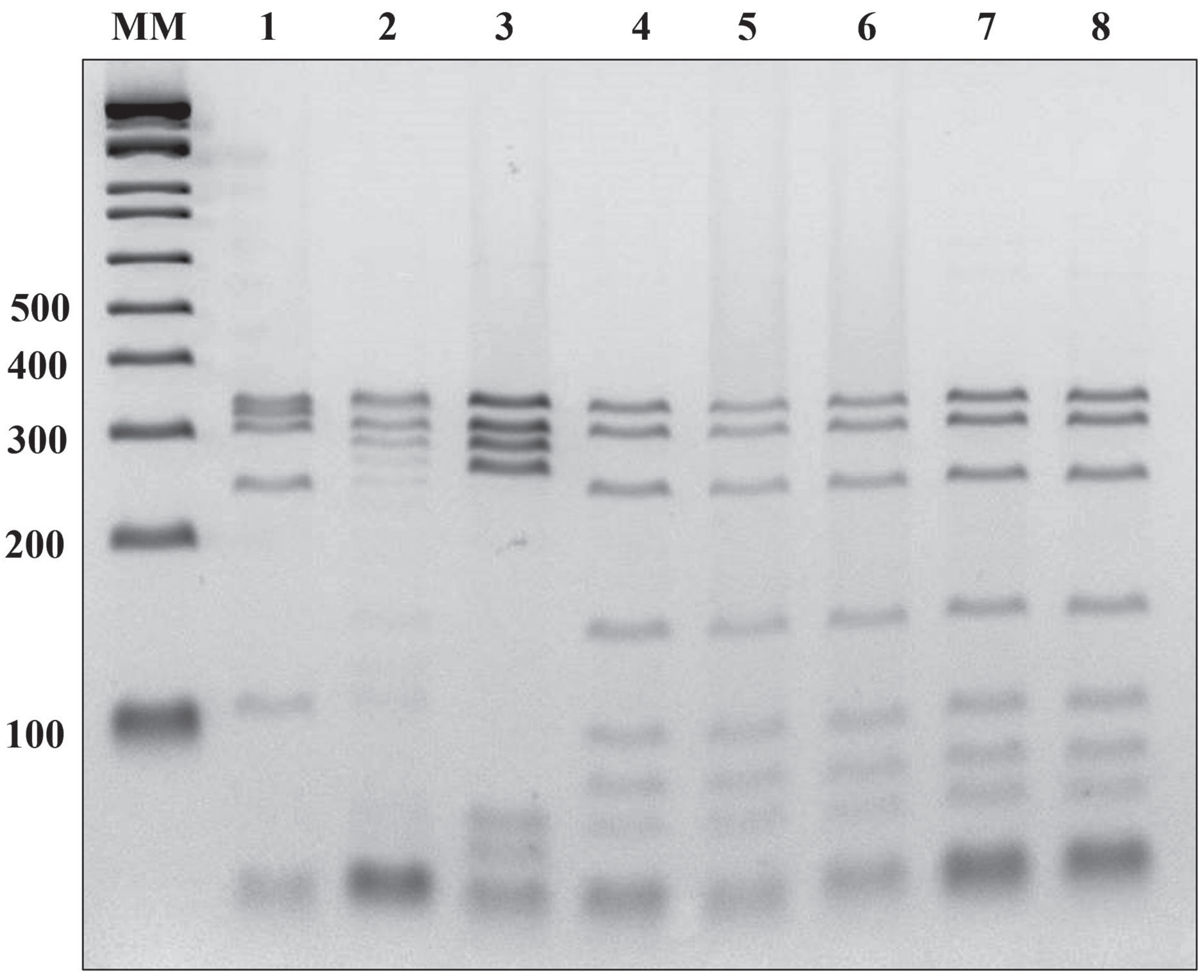

Representative 2% agarose gel of molecular typing of *Leishmania infantum* strains targeting *hsp70* polymerase chain reaction-restriction fragment lenght polymorphism (PCR-RFLP). Lanes: MM, molecular weight marker (1 kb); 1, *L. amazonensis* (IFLA/BR/1967/PH8); 2, *L. braziliensis* (MHOM/BR/1975/M2903); 3, *L. guyanensis* (MHOM/BR/1975/M4147); 4, *L. infantum* (MHOM/BR/1974/PP75); 5-8, *L. infantum* strains (HP-EMO, CUR 268, Ba262 and BH46) used in this study.
